# Multimode Fano Resonances Sensing Based on a Non-Through MIM Waveguide with a Square Split-Ring Resonance Cavity

**DOI:** 10.3390/bios12050306

**Published:** 2022-05-06

**Authors:** Jianfeng Chen, Xinyu Lian, Ming Zhao, Chenbo Xie

**Affiliations:** 1 Key Laboratory of Atmospheric Optics, Anhui Institute of Optics and Fine Mechanics, Hefei Institutes of Physical Science, Chinese Academy of Sciences, Hefei 230031, China; jianfengchen1212@mail.ustc.edu.cn (J.C.); zhaom@aiofm.ac.cn (M.Z.); 2 University of Science and Technology of China, Hefei 230026, China; 3 Advanced Laser Technology Laboratory of Anhui Province, Hefei 230037, China; 4 School of Instrument Science and Optoelectronic Engineering, Hefei University of Technology, Hefei 230009, China; lian@mail.hfut.edu.cn

**Keywords:** non-through MIM waveguide, fivefold Fano resonances, independent tuning, refractive index sensing

## Abstract

In this article, a non-through metal–insulator–metal (MIM) waveguide that can excite fivefold Fano resonances is reported. The Fano resonances are obtained by the interaction between the modes excited by the square split-ring resonator (SSRC) and the bus waveguide. After a detailed analysis of the transmission characteristics and magnetic field strength of the structure using the finite element method (FEM), it was found that the independent tuning of Fano resonance wavelength and transmittance can be achieved by adjusting the geometric parameters of SSRC. In addition, after optimizing the geometric parameters, the refractive index sensing sensitivity (S) and figure of merit (FOM) of the structure can be optimal, which are 1290.2 nm/RIU and 3.6 × 10^4^, respectively. Additionally, the annular cavity of the MIM waveguide structure can also be filled with biomass solution to act as a biosensor. On this basis, the structure can be produced for optical refractive index sensing in the biological, micro and nano fields.

## 1. Introduction

For half a century since 1961, Fano resonance has proven to be one of the important cores in the development of the field of optical sensing. Unlike the traditional Lorentz line shape, the Fano resonance excited by the mutual coupling of discrete and continuous states in the system exhibits a sharp asymmetric line shape [1,2,3]. Therefore, systems that excite Fano resonances are very sensitive to the medium environment in a specific frequency range, exhibiting faster optical responses and high local field enhancement. This makes it very suitable to be used in refractive index sensors [4,5,6], slow light devices [7,8,9,10], interferometers [11,12,13,14] and other fields. In the subwavelength range, a type of surface plasmon polaritons (SPPs) generated by incident photons of free electron-coupled devices at metal interfaces is not limited by the diffraction limit of conventional optics [15,16,17]. Benefiting from this, the realization of modern micro-nano on-chip optical devices is inseparable from the combination of SPPs and Fano resonance. Metal–insulator–metal (MIM) waveguides stand out among many waveguide devices that can realize nanoscale optical information transmission due to their lightweight structure and low cost. It is usually designed for filters [18,19], wavelength division multiplexers [20,21], sensors [22,23,24] and all-optical switches [25,26,27], etc. In these applications, MIM optical waveguide structures that can excite Fano resonances have attracted great interest from researchers [28,29,30]. By way of example, an artificially introduced U-shaped tunnel-side coupled double nanoring resonator cavity waveguide was proposed by Wei et al., where the symmetric Lorentz line shape was transformed into an asymmetric Fano resonance in the original resonance spectral range [31]. Qi et al. proposed an asymmetric MIM structure in which the coupling between the internal bus waveguide and the rectangular cavity excites double Fano resonances [32]. A MIM waveguide consisting of the coupling of a side-coupled rectangular cavity (SCRC), a rightward opening semi-ring cavity (ROSRC) and a silver-air-silver barrier (SASB) was designed by Liu et al. The triple Fano resonance is independently tuned by the parameters of ROSRC for use in biosensors [33]. A MIM waveguide consisting of a circular split-ring resonance cavity (CSRRC) and a double symmetric rectangular stub waveguide (DSRSW) is designed by Chen et al., which can excite quadruple Fano resonances [34]. A plasmonic structure of a side-coupled split-ring resonator by controlling the split-ring direction to tune the Fano resonance wavelength and linear shape was designed by Sun et al. [35]. The various above-mentioned structures that can excite double and multiple Fano resonances are undoubted of great significance to the development of modern optical sensors. Therefore, the flexible tunability of multiple Fano resonances excited by multi-geometric MIM waveguide structures needs to be discussed in more detail. At the same time, the proposal of simultaneous detection of different biomass solutions by multiple ring cavities will lead to the development of optical biosensors.

In this article, a non-through MIM waveguide that can excite fivefold Fano resonances, which consists of a square split-ring resonator (SSRC) and a bus waveguide, is designed. The FEM was used to analyze the mechanism of Fano resonance formation in the structure and the distribution of magnetic field strength at the resonance wavelength. The effects of refractive index change and geometric parameters such as opening width and orientation of SSRC on Fano resonance are used to study the flexible tunability of Fano resonance. In addition, after optimizing the geometric parameters, better sensitivity and FOM are obtained by the structure, which is very suitable for optical sensing in the subwavelength range. In addition, the structure also exhibits excellent optical biosensing properties, which can be used to detect the concentration of biomass solutions.

## 2. Materials and Methods

**Figure 1:**
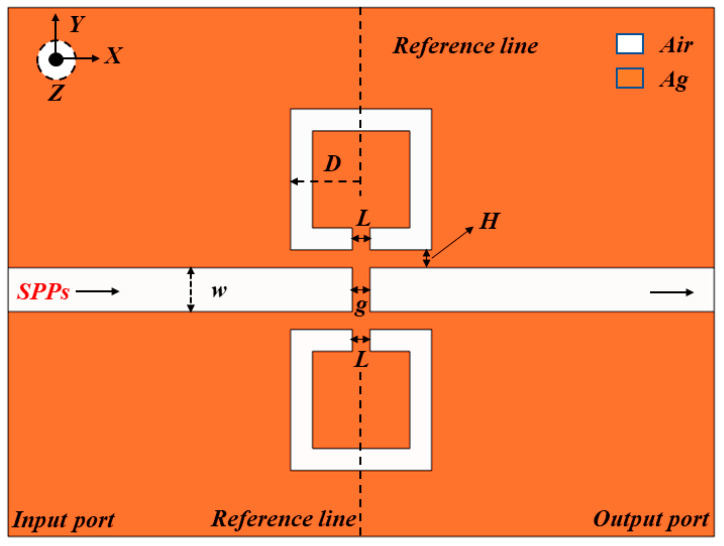
A 2D schematic diagram of a MIM waveguide with double symmetric SSRC.

The MIM waveguide structure formed by an SSRC and a bus waveguide was geometrically modeled using COMSOL Multiphysics 5.6, as shown in Figure 1. To optimize the calculations, a 2D structure was used instead of a 3D structure, which differs by a very small amount because the Z-axis of the metal is much larger than the wavelength of light. The overall structure is symmetrical according to the reference line, in which silver and air are represented in orange and white, respectively.

Silver is chosen as the filler layer due to its low relative permittivity in electromagnetic response. This not only reduces its own power consumption but also ensures that the structure can obtain a strong electric field strength. The relative permittivity of silver is usually defined using the Debye–Drude dispersion model [36,37]:

(1) $$\varepsilon \left(\omega \right)=\varepsilon_{\infty }+\frac{\varepsilon_{s}-\varepsilon_{\infty }}{1+i\tau \omega }+\frac{\sigma }{i\omega \varepsilon_{0}},$$

where *ε_s_* *=* −9530.5, *ε_∞_* = 3.8344, *σ* = 1.1486 × 10^7^ s/m, *τ* = 7.35 × 10^−15^ s, *ω*, which represent the static permittivity, the infinite permittivity, the conductivity of silver, the relaxation time, and the angular frequency of the input wave, respectively. The relative dielectric constant of air *ε_d_* = 1. *L*, *D*, *H*, and *g* represent the SSRC opening width, effective radius, coupling height between modes, and silver baffle width, respectively. Due to the nanoscale geometry, low fabrication precision can lead to deviations of geometric parameters from predetermined values. Therefore, how to realize this structure using the existing manufacturing technology needs to be briefly explained. Among the widely used techniques are electron beam lithography (EBL), focused ion beam (FIB), and nanoimprint lithography (NIL). However, these technologies have their own advantages and disadvantages. The first two are time-consuming and expensive to manufacture but have high precision, while the latter is less expensive and suitable for mass production but has low precision. In order to guarantee the high precision of the designed structure, the Ag filling layer is usually prepared by chemical vapor deposition on the silicon substrate, and then the SSRC and bus waveguides are etched on it by FIB. Therefore, the Ag filling layer is usually prepared by chemical vapor deposition on a silicon substrate. Then, the SSRC and bus waveguides are etched on it by a focused ion beam.

In the Fano resonance, discrete states with almost zero dipole moment cannot couple efficiently with incident light waves. Only on the premise that the wavelength conforms to the resonance condition, will there be stable standing wave transmission in the waveguide structure. The resonance wavelength is usually determined by the following formula [37,38]:

(2) $$\lambda =\frac{2Re(n_{eff})l_{1}}{m-\phi /2\pi },\;m=1,\;2,\;3\;\ldots ,$$

where *ϕ*, *m*, *l*_1_, and *Re*(_neff_) represent the phase shift of the light reflection, the modal order of the resonance, the effective length of the cavity, and the real part of the effective refractive index, respectively. In the simulation modeling process, these media and structural parameters are designed by a perfectly matched layer (PML). When solving a wave electromagnetic field problem, it is usually necessary to simulate a domain with open boundaries, meaning that the boundaries of the computed domain support the passage of electromagnetic waves in a reflection-free manner. Mathematically speaking, PML is just a domain with anisotropy and complex-valued permittivity and permeability. Although the PML is theoretically free of reflections, due to the numerical discretization of the mesh, it still exhibits some reflections when the wave propagates almost parallel to the boundary. Fortunately, similar scenarios are rare in practice. Another characteristic of PML is that it absorbs not only radiative waves but also evanescent waves. Therefore, from a physical point of view, PML can indeed act as a material layer with perfect absorption characteristics. The transmittance from the input (*P_a_*) to the output (*P_b_*) is expressed as *T* = (*S*_21_)^2^, where *S*_21_ represents the transmittance coefficient. A light source that can be coupled into a single-mode fiber generates electromagnetic waves at *P_a_* to excite SPPs. It should be noted that, because the energy and wavelength band of ordinary light sources are difficult to meet the requirements, the preferred light source is usually a laser. The data in Table 1 were combined with the FEM method to calculate and analyze the optical transmission properties of the structure. It should be noted that only the transverse magnetic mode (TM_0_)) should exist in the incoming SPPs wave, so the width of the bus waveguide should be fixed to 50 nm.

## 3. Simulations and Results

### 3.1. Resonance Mechanism and Magnetic Field Distribution

**Figure 2:**
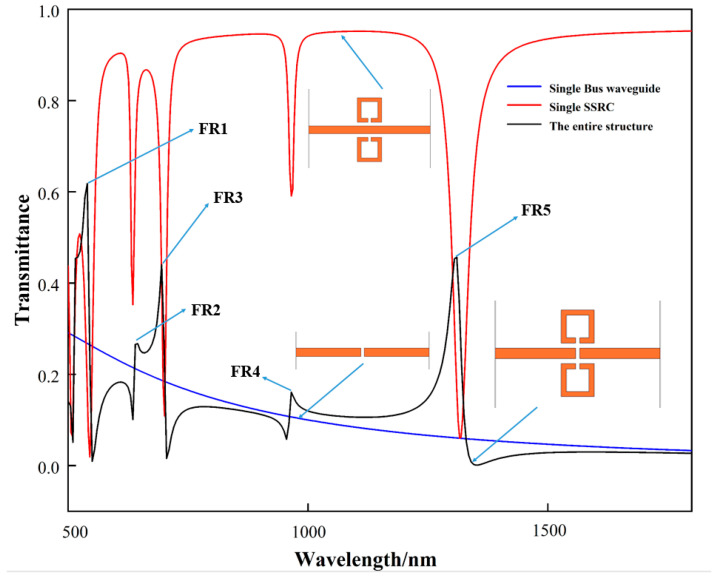
Schematic diagram of the formation mechanism of Fano resonance excited by the designed structure.

First, the coupling mechanism between single SSRC, single bus waveguide and the entire structure is analyzed to illustrate the superior sensing characteristics of the structure, as shown in Figure 2. The structures were set to single SSRC mode, single bus waveguide mode and full structure mode, respectively, and the transmission lines were calculated by inputting the same laser source. It should be noted that the transmission spectra of the three are independent. Still, in order to explain the formation mechanism of Fano resonance more intuitively, the three spectral lines are presented in Figure 2 at the same time. As indicated by the blue line, its profile is almost horizontal and the transmittance is always between 0.18 and 0.35. In other words, the wide-band continuous state with an extremely wide spectral width is excited by the bus waveguide. As shown in the red line, the spectral lines have five dips, and the transmittance is as low as 0.12. Therefore, it can be considered that the narrow-band discrete state with an extremely narrow spectral width is excited by the single SSRC. As shown in the black line, the full structure has five rapidly declining and asymmetric profiles, which are completely different from the Lorentz lines. This also indicates that the fivefold Fano resonances are excited by the mutual interference of the narrow-band discrete state and wide-band continuous state between the entire structures. Since Fano resonance includes both resonance peaks and resonance valleys, in order to facilitate quantitative analysis of the properties of the spectrum, five resonance peaks are defined as FR1, FR2, FR3, FR4 and FR5, respectively.

**Figure 3:**
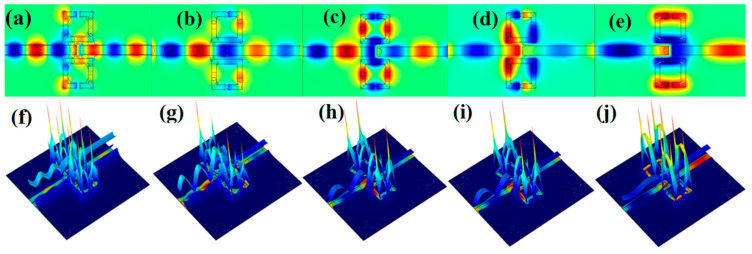
(**a**–**j**) The 2D and 3D distributions of magnetic field intensity (|*Hz*|*^2^*) at λ = 542 nm, 650 nm, 698 nm, 968 nm and 1313 nm.

The magnetic field strength (|*Hz*|^2^) of the fivefold Fano resonances was investigated to further analyze its resonance mechanism in the structure, as shown in Figure 3. The magnetic field energy distribution of the whole structure is basically symmetrical to the bus waveguide. It can be seen from Figure 3a–j that most of the energy of SPPs can only propagate in the bus waveguide and SSRC on the surface of the quartz substrate. This is due to the fact that SPPs only support propagation at the interface of metal and dielectric, not the interface of two dielectrics. At the same time, the energy of SPPs is mostly concentrated in the SSRC, which can be transmitted to the output port only when the structure is at the resonance wavelength; otherwise, it cannot be transmitted. This can actually be understood as the fact that the two transmission paths of SPPs determine this phenomenon. One is that the SPPs enter the bus waveguide directly from P1 to P2 to generate a broadband continuum. The other is to enter SSRC first and then transmit to P2, resulting in a narrow-band discrete state. The states produced by these two paths interfere at P2 to form a standing wave. Furthermore, it can be seen from Figure 3f–j that the SPPs in SSRC increase significantly when the structure is at the resonance wavelength, the interference between modes is enhanced, and the structure thus captures the ultra-low transmittance.

### 3.2. Influences of Refractive Index and Geometric Parameters on Resonances

**Figure 4:**
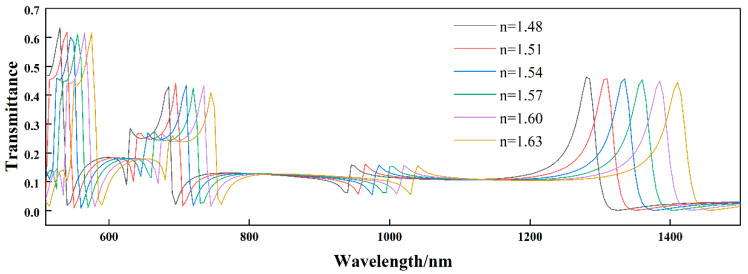
Influence of different refractive indices on the structural Fano resonances.

The above analysis shows that the entire waveguide structure excites the Fano resonance when at the resonance wavelength. At the same time, the asymmetric line shape of Fano resonance also makes its resonance wavelength extremely sensitive to the change in refractive index [34]. Furthermore, the ultra-narrow transmission peaks generated by Fano resonance can significantly improve the sensing resolution. Therefore, the effect of the refractive index of the medium on the transmission properties of the structure should be analyzed in more detail. By changing the refractive index of the medium from 1.48 to 1.63 in steps of 0.03, as shown in Figure 4, it can be seen that with the increase of the refractive index, the five Fano resonance peaks have obvious red shifts. Still, the profiles of resonances are not affected. As a specific physical quantity in optics, the refractive indices of media are usually different. Therefore, some biological media such as interstitial fluid and hemoglobin can be filled into SSRC, and the relevant properties such as concentration can be obtained by detecting the change of its refractive index.

**Figure 5:**
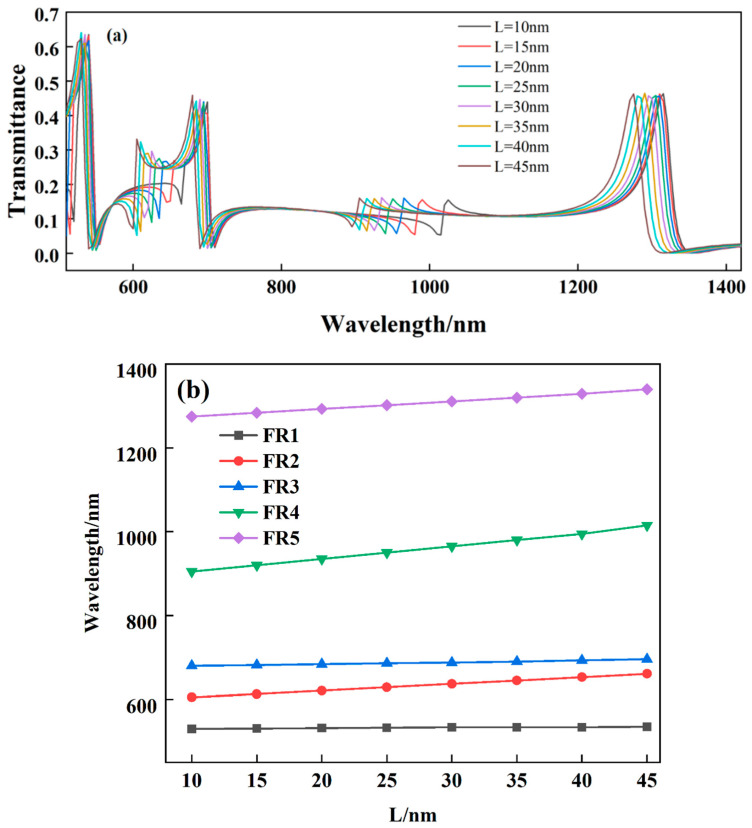
(**a**) Influence of different L on the structural Fano resonances. (**b**) Fano resonance wavelengths of structures at different L.

As can be seen in Figure 3, when the structure is at the resonant wavelength, the magnetic field energy is almost entirely concentrated in the SSRC. We speculate that the extreme sensitivity of the Fano resonance wavelength to the geometrical parameters of the SSRC leads to this. To verify this, the effect of different opening widths of the SSRC on the structural Fano resonance was investigated, as shown in Figure 5. It can be seen from Figure 5a that with the gradual increase of the opening width (also known as the effective radius), FR1 and FR3 undergo a slight redshift, and FR2, FR4, and FR5 undergo a significant redshift. As can be seen in Figure 5b, the red shifts of the five Fano resonance peaks are linearly related to the amount of opening width variation of the SSRC. Intuitively, the opening width of the SSRC can determine the specific position of the Fano resonance wavelength, which also means that the fivefold resonances of the resonator can be independently tuned by the narrow-band discrete states. Therefore, the structure can be used as a tunable optical sensor in optics, biology, medicine and other fields.

**Figure 6:**
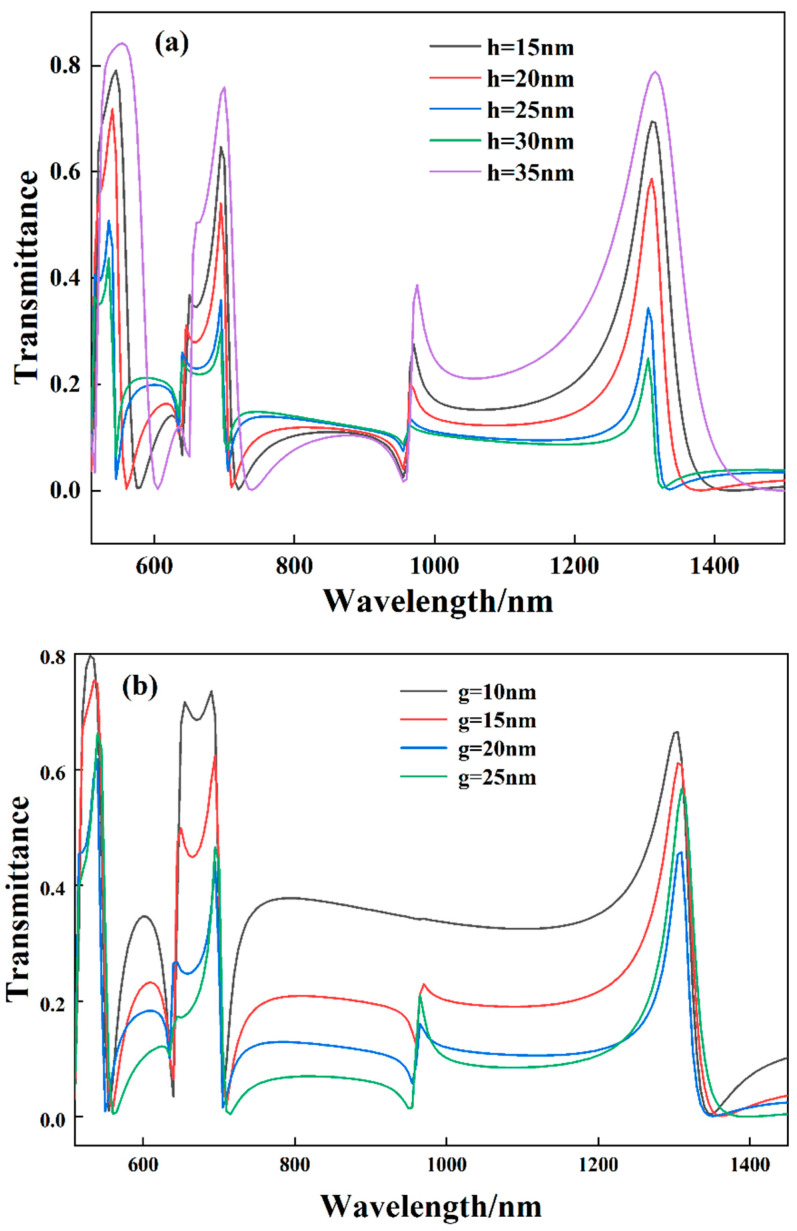
(**a**) Influences of different coupling heights between modes on structural Fano resonances. (**b**) Effect of different silver baffle widths on structural Fano resonances.

In the production and processing of modern precision instruments, the finished product usually cannot be processed twice. Therefore, the geometrical parameters of the structure should be discussed in order to fabricate the sensor with optimal performance. The effects of coupling distance and silver baffle width on the structurally excited Fano resonance are discussed. First, the coupling distance was increased from 15 nm to 35 nm in steps of 5 nm, as shown in Figure 6a. With the continuous change of the coupling distance, the transmittance of the Fano resonance has a significant increase and decrease. Still, the resonance wavelength is basically unchanged, and the profile is roughly the same. This is due to the weakening of the coupling between the narrow-band discrete state and the wide-band continuous state excited by the SSRC as it moves away from the bus waveguide. Next, the effect of the variation of the silver baffle width on the structural Fano resonance is investigated, as shown in Figure 6b. Much like the change in coupling height, the transmittance of the spectrum decreases as the width of the silver baffle increases, but the resonant wavelength remains essentially unchanged.

**Figure 7:**
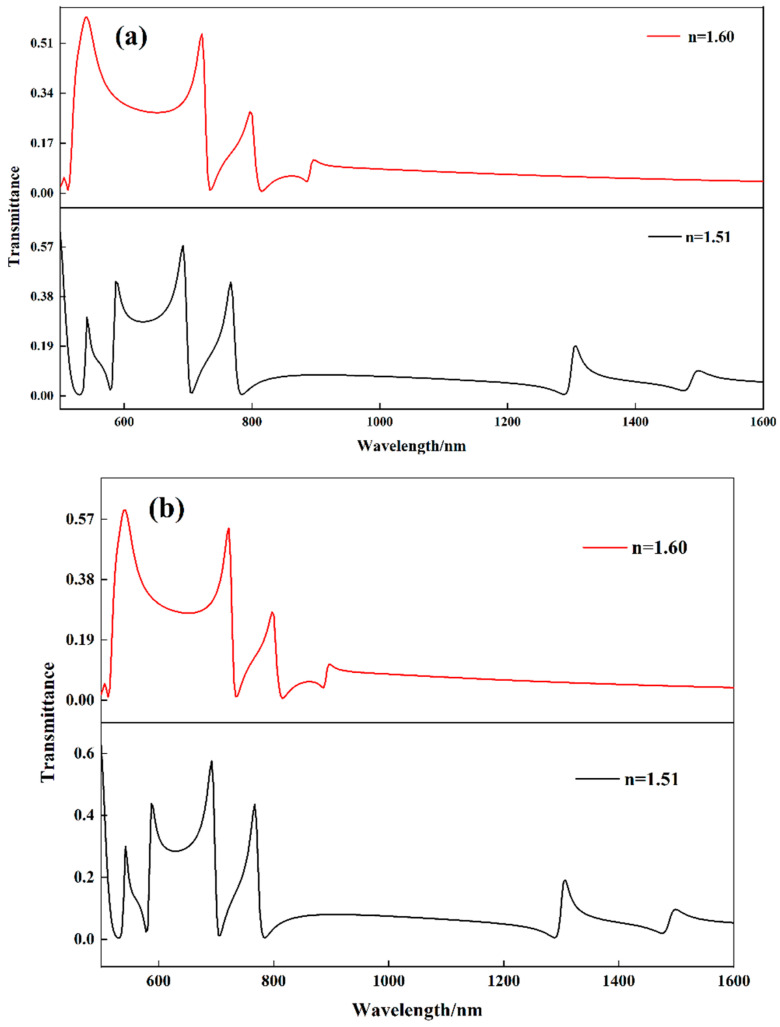
Influences of different SSRC opening directions on the structural Fano resonances (**a**) upper edge and (**b**) all-around.

Since SSRC is not a generally circular ring cavity but a square ring cavity, the effect of the opening direction on Fano resonance should be discussed. First, the opening was changed to the position of the upper side of the SSRC, and the transmission spectra of the structure with different refractive indices were studied, as shown in Figure 7a. With the increase of the refractive index, the Fano resonance decreased from the original five resonance peaks to three, and the spectral transmittance decreased significantly. Next, the effect of the four-sided opening of the SSRC on the structural Fano resonance is investigated, as shown in Figure 7b. It can be seen that it is almost consistent with the change of the lower opening; with the increase of the refractive index, the number of Fano resonances peaks decreases to three, and the transmittance decreases significantly. After changing the opening position, it can be seen that the coupling between the two modes excited by the structure is significantly weakened, which is undoubtedly unfavorable for optical sensing. It also shows that the original structure proposed in the manuscript can be regarded as having optimal geometric parameters.

### 3.3. Structural Sensitivity and FOM

**Figure 8:**
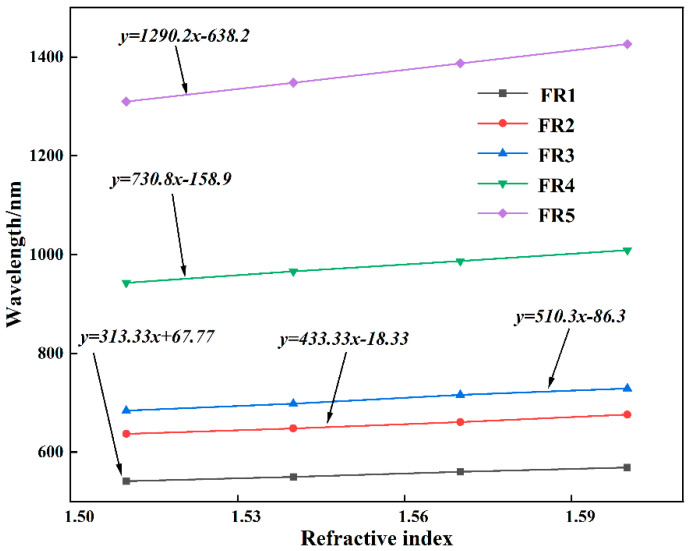
Refractive index sensing sensitivity of fivefold Fano resonances.

From the above discussion, it can be seen that the change in refractive index is extremely sensitive to the Fano resonance excited by the structure. Therefore, the sensitivity can be used to quantitatively represent the refractive index sensing properties of the structural Fano resonance. It is usually defined as the offset change between the refractive index (Δ*n*) and the resonant wavelength (Δ*λ*), i.e., *S* = Δ*λ/*Δ*n* [39]. Specifically, the relationship between the resonance wavelengths of the fivefold Fano resonance peaks excited by the structure and the refractive index change is studied, as shown in Figure 8. It can be seen that the fivefold Fano resonance peaks have good linearity under different refractive index changes, and the linear correlation coefficients are 0.9995, 0.9997, 0.9998, 0.9999 and 0.9996, respectively. Therefore, the sensitivities of the structures at different resonance wavelengths are 313.33 nm/RIU, 433.33 nm/RIU, 510.3 nm/RIU, 730.8 nm/RIU and 1290.2 nm/RIU, respectively. In addition, the structural sensitivities of some similar works are presented in Table 2 for comparison with the structure of this manuscript. It can be seen that among structures that can excite multiple Fano resonances, the present structure achieves moderate sensitivity. It should be mentioned that the geometric parameters such as the opening width and coupling distance of the SSRC are tuned in steps of 5 nm. At the same time, the structure is etched on a substrate with a length of 600 nm and a width of 500 nm, and the adjustable range of geometric parameters is much smaller than the size of the substrate. Therefore, the structure can accept a minimum tuning size of 5 nm and a tuning interval of 30 nm during fabrication. This also means that nano-level manufacturing precision puts forward higher requirements for modern industrial manufacturing technology.

**Figure 9:**
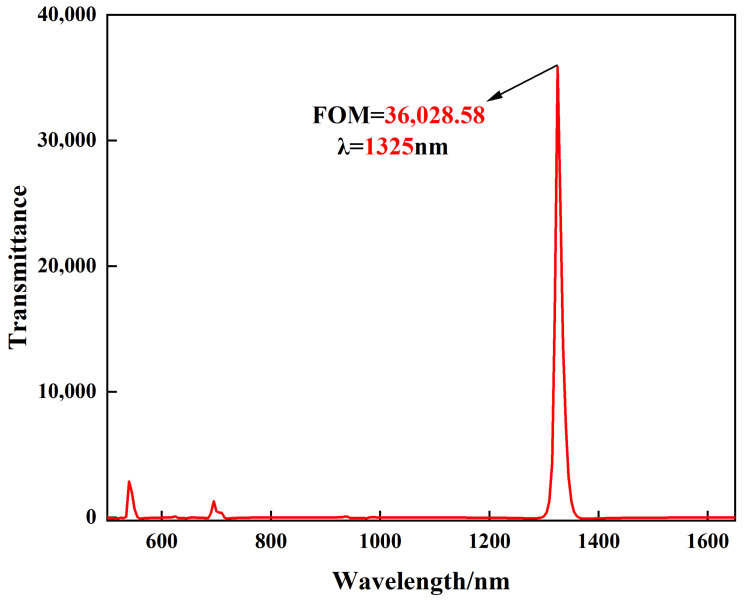
The FOM value of the structure.

In addition, the sensitivity alone cannot be a strong indicator of the sensing performance, so another key factor, the FOM, is introduced to evaluate the sensing performance of the structure. This is usually defined as *FOM* = Δ*T*/(*T*·Δ*n*), where *T* is the transmission of the structure, and Δ*T* is the relative transmittance change at a fixed wavelength caused by the refractive index change (Δ*n*) [44]. As shown in Figure 9, FOM up to 3.6 × 10^4^ is obtained by this structure due to the ultra-low transmittance of the FR5 dip at wavelength λ = 1325 nm.

### 3.4. Structural Biosensing Application

**Figure 10:**
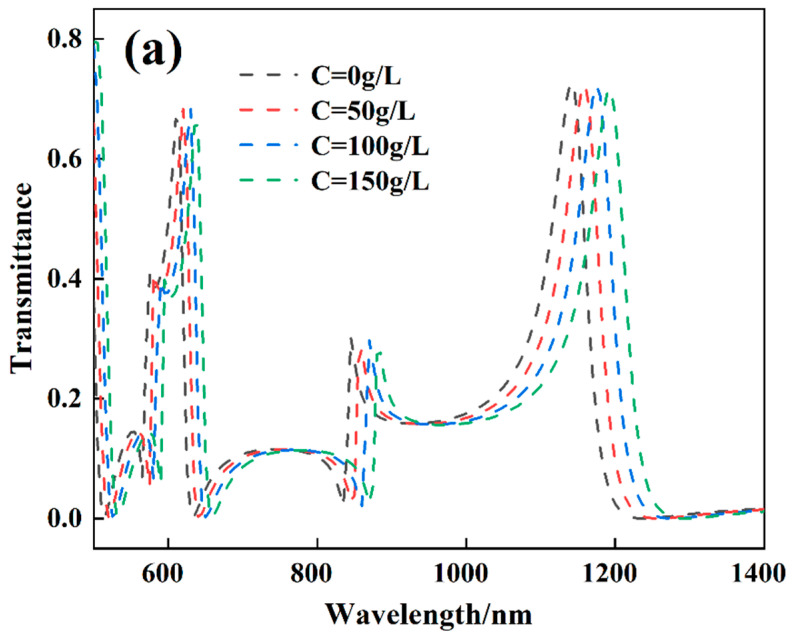

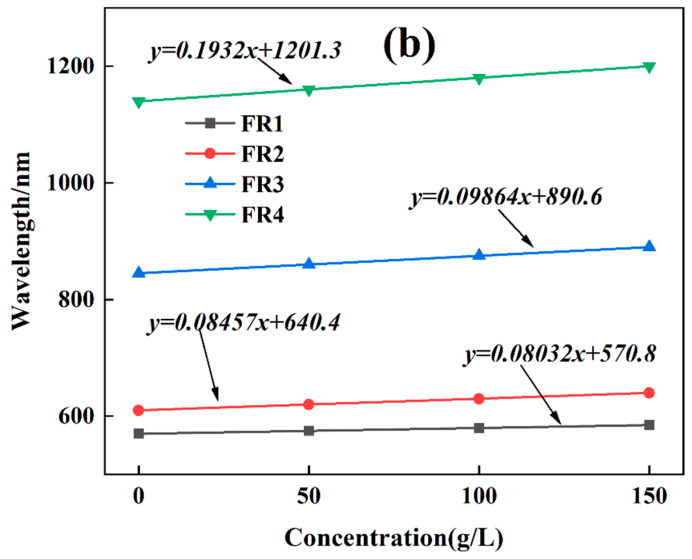
(**a**) Transmission spectra of Fano resonances at different glucose solution concentrations. (**b**) Relationship between quadruple Fano resonances wavelength and glucose solution concentration.

Optical biosensing has become a research hotspot in recent years. Next, we determined whether the designed structure is suitable for use in biosensors by measuring the concentration and sensitivity of glucose. In biomedicine, glucose solutions are used to treat undereating or massive fluid loss. The refractive index of its solution is usually described by *n* = 0.00011889 *C* + 0.31230545, where *n* and *C* are the refractive index and concentration of glucose concentration, respectively [44,45]. Therefore, the Fano resonances transmission spectra at different glucose solution concentrations were considered. It can be seen from Figure 10a that after the ring cavity is filled with glucose solution, the structure obtains quadruple Fano resonance peaks, and the transmission spectrum has an obvious red shift as the concentration changes from 0 g/L to 150 g/L. Figure 10b reflects that the quadruple Fano resonances wavelength has a good linear correlation with the change of glucose solution concentration. These results reveal that the structure is extremely sensitive to changes in biomass solution concentration and can be applied in the field of optical biosensing.

## 4. Conclusions

Overall, a non-through MIM waveguide, which consists of an SSRC and a bus waveguide, is designed. Among them, the fivefold Fano resonances formed by the mutual interference of the two modes excited by the SSRC and the bus waveguide are captured by the structure. In order to design a refraction sensor with optimal performance, the independent tunability of the fivefold Fano resonances transmission characteristics is discussed in detail. The results show that the specific number and position of the Fano resonances wavelength can be independently tuned by the opening width and direction of the SSRC flexibly. The transmittance and line shape are regulated by the coupling distance between the two modes and the silver baffle width. After the geometric parameters of the MIM structure are optimized, the sensitivity can reach 1290.2 nm/RIU, and the FOM can reach 3.6 × 10^4^. Meanwhile, the annular cavity of the structure can also be filled with biomass solution to act as a biosensor. In conclusion, the structure can provide a useful reference for the design of optical and biosensors in the subwavelength range.

## Figures and Tables

**Table 1:** Summary of the simulation calculation parameters of the structure.

Parameter	Symbol	Quantity	Unit
The bus waveguide width	*w*	50	nm
Coupling height	*H*	20	nm
Split length of SSRC	*L*	20	nm
Effective Radius	*D*	100	nm
Silver baffle width	*g*	15	nm
Refractive index of bus waveguides	-	1	-
Refractive index of SSRC	*n*	1	-

**Table 2:** Comparison of the sensitivity with other references.

Waveguide Structure	Sensitivity	Reference
A half-ring resonator coupled MIM waveguide structure	753 nm/RIU	[40]
MIM waveguide structure consisting of an M-type cavity and a baffle	780 nm/RIU	[41]
An end-coupled ring-groove joint MIM waveguide structure	1050 nm/RIU	[42]
MIM structure with one rectangular and two square nanorod array resonators	1090 nm/RIU	[29]
MIM waveguide consisting of a CSRRC and a DSRSW	1328.8 nm/RIU	[34]
A new racetrack integrated circular cavity based on MIM waveguide	1400 nm/RIU	[43]
This paper	1290.2 nm/RIU	

## Data Availability

Not applicable.
